# Who will benefit from antidepressants in the acute treatment of bipolar depression? A reanalysis of the STEP-BD study by Sachs et al. 2007, using Q-learning

**DOI:** 10.1186/s40345-014-0018-5

**Published:** 2015-04-03

**Authors:** Fan Wu, Eric B Laber, Ilya A Lipkovich, Emanuel Severus

**Affiliations:** Department of Statistics, North Carolina State University, 2311 Stinson Drive, Raleigh, 27695 USA; Quintiles, 4820 Emperor Blvd, Durham, 27703 USA; Department of Psychiatry and Psychotherapy, University Hospital Carl Gustav Carus, Technische Universität Dresden,, Fetscherstraße 74,, 01307 Dresden Germany

**Keywords:** Bipolar disorders, *Q*-learning, Antidepressant, Dynamic treatment regimes

## Abstract

**Background:**

There is substantial uncertainty regarding the efficacy of antidepressants in the treatment of bipolar disorders.

**Methods:**

Traditional randomized controlled trials and statistical methods are not designed to discover if, when, and to whom an intervention should be applied; thus, other methodological approaches are needed that allow for the practice of personalized, evidence-based medicine with patients with bipolar depression.

**Results:**

Dynamic treatment regimes operationalize clinical decision-making as a sequence of decision rules, one per stage of clinical intervention, that map patient information to a recommended treatment. Using data from the acute depression randomized care (RAD) pathway of the Systematic Treatment Enhancement Program for Bipolar Disorder (STEP-BD) study, we estimate an optimal dynamic treatment regime via *Q*-learning.

**Conclusions:**

The estimated optimal treatment regime presents some evidence that patients in the RAD pathway of STEP-BD who experienced a (hypo)manic episode before the depressive episode may do better to forgo adding an antidepressant to a mandatory mood stabilizer.

**Electronic supplementary material:**

The online version of this article (doi:10.1186/s40345-014-0018-5) contains supplementary material, which is available to authorized users.

## Background

Bipolar disorders are a group of chronic lifelong recurrent psychiatric disorders characterized by episodic shifts in mood, energy, social and vocational functioning, and activity levels (Phillips and Kupfer 2013). Worldwide, bipolar disorders are a leading cause of disability (Vos et al. 2013) and associated with a substantial economic burden on society (Kleine-Budde et al. 2014). Standard antidepressant medications have been proved to be effective for acute and long-term treatment of unipolar depression (Bauer et al. 2013); however, supporting evidence for the inclusion of standard antidepressants in the acute and long-term treatment of bipolar depression is more limited and controversial (Grunze et al. 2010, Pacchiarotti et al. 2013). Furthermore, there is concern that antidepressants can induce abnormal mood elevation (Licht et al. 2008). We use data from the Systematic Treatment Enhancement Program for Bipolar Disorder (STEP-BD) (Sache et al. 2003, 2007) to estimate an optimal dynamic treatment regime (DTR) (Chakraborty and Murphy 2014; Murphy 2003, Robins 2004, Schulte et al. 2014), for bipolar depression. A DTR is a sequence of decision rules, one per stage of intervention, that map up-to-date patient information to a recommended treatment; thus, an estimated optimal DTR can be used to generate hypotheses about how patient history and outcomes should dictate treatment selection. The estimated optimal DTR for bipolar depression constructed from the STEP-BD study suggests the hypothesis that standard antidepressants should not be used to supplement mood stabilizers for patients with a prior hypomanic episode.

A DTR aims to select if, when, what, and to whom treatment should be assigned and thereby fits into the paradigm of personalized medicine. Because DTRs select treatment according to the uniquely evolving health status of each patient, they are suited to manage chronic illnesses with patient response heterogeneity; thus, DTRs have tremendous potential for personalizing and improving treatment strategies for bipolar disorder (Leboyer and Kupfer 2010; Nierenberg et al. 2013). Optimal DTRs have been estimated for wide range of chronic illnesses including major depressive disorder (Chakraborty et al. 2013; Chakraborty and Moodie 2013), attention deficit hyperactivity disorder (Laber et al. 2014; Lei et al. 2012, Nahum-Shani et al. 2012a), schizophrenia (Laber et al. 2014; Shortreed et al. 2011), HIV/AIDS (Moodie et al. 2007; Sterne et al. 2009), and cigarette addiction (Strecher et al. 2006). Estimation of an optimal DTR is typically done as a secondary, exploratory analysis and viewed as a method of generating hypotheses for follow-up confirmatory experiments (Murphy ??). This is the perspective we take here; nevertheless, we show that an estimated optimal DTR appears to perform markedly better than any fixed treatment strategy.

In the “STEP-BD study” section, we review the STEP-BD study. In the “Dynamic treatment regimes and *Q*-learning” section, we formalize DTRs and introduce the *Q*-learning estimation algorithm. In the “Analysis of STEP-BD” section, we present an analysis of STEP-BD.

## Methods

The study on which our analyses are based was approved by the institutional review board at each study site and was overseen by a data and safety monitoring board (for more details, see http://www.ncbi.nlm.nih.gov/pubmed/17392295).

### STEP-BD study

**Figure 1:**
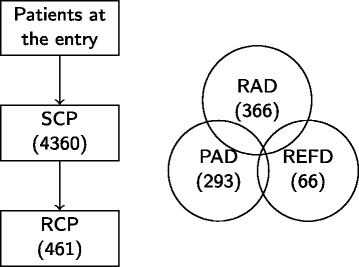
**STEP-BD Design.** There were 4,360 patients who participated in SCP and 461 patients who participated in RCP. Patients in SCP satisfying RCP criteria entered RCP. In RCP, there are three different pathways: RAD, PAD, and REFD. Patients can enter more than one random pathways.

STEP-BD is a long-term study of bipolar disorder funded by the National Institute of Mental Health (NIMH). Its aim was “to generate externally valid answers to treatment effectiveness questions related to bipolar disorder” (Sachs et al. 2003). Patients of age older than 15 years fulfilling DSM-IV criteria for any subtype of bipolar disorders could enter the study registry. In total, 4,360 patients from 22 sites in United States enrolled. The study lasted for 7 years (2001–2007). In STEP-BD, there are two different treatment pathways: standard care pathway (SCP) and randomized care pathway (RCP). SCP is open to all participants with a diagnosis of bipolar disorders. Each treatment delivered is open and will follow treatment guidelines. Decisions are made on the basis of shared decision-making. After patients signed informed consent for entry into the STEP-BD study registry, all patients enter the SCP. If a patient’s status meets the eligibility criteria at one of the follow-up visits during the SCP for a study within the RCP, additional consent is requested for entry into that RCP. The RCP utilizes methods appropriate for efficacy studies, and random assignment is needed to provide answers to clinical questions. In the RCP, there are three different pathways each addressing unmet needs in the treatment of bipolar disorder: acute depression randomized (RAD) pathway, acute depression psychosocial intervention (PAD) pathway, and refractory depression (REFD) pathway. If patients are unwilling to consent to one of the RCPs, they remain in the SCP. In general, the decision of pathway (SCP versus RCP) is based on both the doctor’s and patient’s opinion. In STEP-BD, patients could switch pathways based on doctor’s or their own preference as well as inclusion and exclusion criteria. Figure 1 shows the diagram of STEP-BD study. Our analysis utilizes patients enrolled in RAD.

### Acute depression randomized pathway

As mentioned above, the RAD pathway is one of the three RCPs in STEP-BD. In addition to satisfying the general entry criteria for STEP-BD study registry, patients had to be at least 18 years old and fulfill the DSM-IV criteria for a major depressive episode in the context of bipolar I or bipolar II disorder. All patients with a history of intolerance or non-response to both bupropion and paroxetine were excluded, as well as those requiring current short-term treatment for a coexisting substance-abuse disorder or requiring the addition of antipsychotic medication or a change in the dose of a long-term antipsychotic medication (Sachs et al. 2007). In addition, patients had to take a mood stabilizer at the time of randomization or agree to begin treatment with a mood stabilizer. Moreover, they had to agree to have all non-study antidepressants tapered after initiation of study drug, with the antidepressant discontinued by the end of week 2. The purpose of RAD was to explore the effectiveness of adjunctive antidepressant treatment, in addition to a mood stabilizer. Initially, the mood stabilizers were limited to lithium, valproate, the combination of lithium and valproate, or carbamazepine. However, later on, any FDA-approved antimanic agent could be used as mood stabilizers. Table 1 shows the percentages of different mood stabilizers used in RAD. At week 0, patients were randomly assigned to one antidepressant (150 mg of a sustained release formulation of bupropion or 10 mg of paroxetine to begin with) or placebo. After 6 weeks, patients with non-response on the placebo were randomized to either paroxetine or bupropipon; patients with non-response on the antidepressant were assigned to either openly increase the dose of their current antidepressant or add another antidepressant. At weeks 8, 10, or 12, clinicians will make final decision for patients based on their clinical status collected from clinical monitoring form (CMF).

**Table 1:** **Percentages of different mood stabilizers used in RAD**

**Mood stabilizer**	**Percentage (%)**
Aripiprazole	2.19
Carbamazepine	6.28
Clozapine	0.27
Lithium	48.91
Olanzapine	9.84
Quetiapine	9.02
Risperidone	7.10
Valproate	41.53
Ziprasidone	3.01

Note that some patients received more than one mood stabilizer.

**Figure 2:**
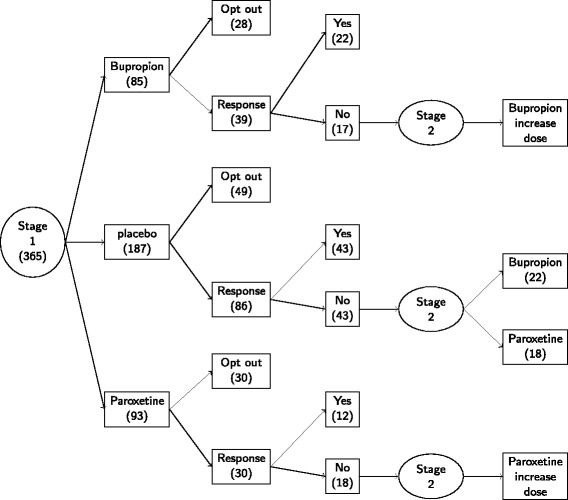
**RAD design.** At the beginning (stage 1), there are 365 patients in total. Eighty-five patients take Bupropion, 93 patients take Paroxetine, and 187 patients take placebo. After 6 weeks, 104 patients’ information are lost. Only 78 patients are tracked with non-response at the end of stage 1. At stage 2, patients with non-response are assigned to secondary treatment intervention. Patients taking Bupropion or Paroxetine at stage 1 will increase current doses. But patients taking placebo at stage 1 will be assigned Bupropion or Paroxetine.

During the study, patients need to visit their doctors every week to fill in the CMF (Sachs et al. 2002). Patients were allowed to switch to SCP (opt out) at any time by their preference or doctor’s opinion. Patients who had severe adverse effects or met criteria for hypomania or mania discontinued the antidepressant or placebo and received open treatment while remaining in STEP-BD. Since after 6 weeks, only one patient with non-response on the antidepressant was assigned to add another antidepressant, we ignored this one observation and supposed patients with non-response to antidepressant after 6 weeks were only assigned to increase the dose of their current antidepressant. Figure 2 shows a schematic for the RAD protocol. Response for a given subject was defined as at least 50*%* improvement over their initial SUMD (scale scores for depression) and not meeting the DSM-IV criteria for hypomania or mania. Scores on the continuous symptom subscale for depression (SUMD) range from 0 to 22, with higher scores indicating more severe symptoms. Both SUMD and SUMM (symptom subscale for mood elevation, SUMM scores range from 0 to 16) are part of the modified clinical monitoring form for mood disorders (Sachs et al. 2002). Because subjects in RAD are potentially randomized multiple times with randomizations occurring at crucial points of the disease process, RAD is an example of a Sequential Multiple Assignment Randomized Trial (SMART) (Lavori and Dawson 2004; Murphy 2003). Data collected in SMARTs can be used to efficiently estimate and evaluate DTRs. In the next section, we formalize the notion of an optimal DTR and introduce a regression-based approach called *Q*-learning for estimating an optimal DTR from a SMART.

### Dynamic treatment regimes and *Q*-learning

The effective management of a chronic illness requires ongoing personalized treatment (Wagner et al. 2001). DTRs formalize clinical decision-making as sequence of *decision rules*, one per treatment decision, that map patient information to a recommended treatment. An optimal DTR yields the optimal expected outcome when applied to assign treatment to a population of interest. One method for estimating an optimal DTR from observational or randomized study data is *Q*-learning (Murphy ??; Schulte et al. 2014). *Q*-learning is an approximate dynamic programming algorithm that can be viewed as an extension of regression to multi-stage decision problems (Nahum-Shani et al. 2012b). As our focus is the application of *Q*-learning to the RAD study within the RCP pathway, we focus on data from a two-stage randomized trial with a terminal continuous outcome; however, *Q*-learning applies in much more general settings (Goldberg and Kosorok 2012; Laber et al. 2014; Moodie et al. 2014; Schulte et al. 2014; Sutton and Barto 1998; Watkins and Dayan 1992).

*Q*-learning estimates an optimal regime using backward induction. For simplicity, we assume that the entire treatment period contains two stages with a distal outcome measured after completion of the second stage; treatment decisions are made in the beginning of each stage. *Q*-learning proceeds in two steps. In the first step, it estimates an optimal treatment rule for the second stage of treatment given patient-level data accumulated up to and immediately preceding this second treatment assignment. This information includes each patient’s baseline information, stage 1 treatment assignment and intermediate, i.e., proximal, outcomes measured during the course of the first stage of treatment. These inputs to the second-stage rule are treated as “independent variables” with no attempt to infer what decision at stage 1 would be optimal for a given patient. This first step is achieved by regressing the distal outcome on patient information up to decision stage 2 and manipulating the obtained analytic expression to find for each patient which treatment at stage 2 optimizes the expected distal outcome.

At the second step, *Q*-learning looks for treatment assignment at stage 1 that would result in optimal distal outcome, assuming that subsequent stage 2 treatment will be determined by the rule constructed in step 1 of the procedure. Such backward reasoning allows *Q*-learning to factor in future decisions when making treatment decisions at earlier stages. This can be contrasted with a myopic strategy that only looks at intermediate (proximal) outcomes of a current treatment assignment. For example, treatments at stage 1 may lead to temporary alleviation of symptoms and therefore appear beneficial for a patient; however, the long-term benefits may become questionable after the later (e.g., second) stage decisions are factored in.

### Formal mathematical description of *Q*-learning

We now present formal mathematical description of *Q*-learning. We assume that data available to estimate a DTR are in the form of *n* independent, identically distributed trajectories $\lbrace (X_{1i}, A_{1i}, X_{2i}, A_{2i}, Y_{i}) \rbrace _{i=1}^{n}$, one for each subject where: $X_{1} \in \mathbb {R}^{p_{1}}$ denotes baseline (pre-randomization) subject information; $A_{1} \in \mathcal {A}_{1}$ denotes the first-stage treatment assignment; $X_{2} \in \mathbb {R}^{p_{2}}$ denotes information collected during the course of the first-stage treatment including information dictating first-stage responder status; $A_{2} \in \mathcal {A}_{2}$ denotes the second-stage treatment assignment; and $Y\in \mathbb {R}$ denotes a continuous outcome measured at the end of the study coded so that lower values are better. To match the RAD study, we assume that responders are not re-randomized. In the RAD study, *X*_1_ contains a subject’s age, race, gender, marital status, annual household income, employment status, education level, nine different side effect measures, medical insurance type, as well as baseline measures of bipolar type, clinical status prior to depressive episode, scale scores for mood elevation (SUMM), and scale scores for depression (SUMD); *A*_1_ denotes low-dose Bupropion, low-dose Paroxetine, or placebo; *X*_2_ contains responder status at the end of stage 1, as well as SUMM and SUMD at the end of stage 1; *A*_2_ denotes either high-dose Bupropion or high-dose Paroxetine; *Y* is SUMD measured at the end of stage 2.

We use uppercase letters to denote random variables and lowercase letters to denote instances of these random variables. Define *H*_1_=*X*_1_ and $H_{2} = \left (X_{1}^{\intercal }, A_{1}, X_{2}^{\intercal }\right)^{\intercal }$, so that *H*_*j*_ denotes information available to the decision-maker at stage *j*=1,2. For any random variable *U*, let dom *U* denote the set of possible values for *U*. A DTR is a pair of functions *π*=(*π*_1_,*π*_2_) where *π*_*j*_:dom *H*_*j*_→dom *A*_*j*_ so that a patient presenting with *H*_*j*_=*h*_*j*_ at stage *j* is assigned treatment *π*_*j*_(*h*_*j*_). For any *h*_*j*_∈dom *H*_*j*_, let $\mathcal {F}_{j}(h_{j})$ denote the set of feasible treatments for a patient presenting at stage *j* with *H*_*j*_=*h*_*j*_. In the RAD study $\mathcal {F}_{1}(h_{1}) = \{ \text {Bupropion, Paroxetine, and placebo} \}$, for all *h*_1_. At the second stage, responders are not re-randomized; feasible second-stage treatments for non-responders are:

$$ \mathcal{F}_{2}(h_{2}) \,=\,\! \left\lbrace\!\!\!\begin{array}{ll} \left\lbrace \mathrm{high\text{--}dose\,\,Buproprion}\right\rbrace & \text{if}\,\, A_{1}=\mathrm{\,\,Buproprion}, \\ \left\lbrace \mathrm{high\text{--}dose\,\,Paroxetine}\right\rbrace & \text{if} \,\, A_{1}=\mathrm{\,\,Paroxetine},\\ \left\lbrace \text{Buproprion, Parotexine} \right\rbrace &\text{if}\,\,A_{1}=\text{placebo}. \end{array}\right. $$

Let $\Pi = \lbrace \pi = (\pi _{1}, \pi _{2}),:\, \pi _{j}(h_{j})\in \mathcal {F}_{j}(h_{j})\,, \forall h_{j}\in \text {dom}\,H_{j}\rbrace $ denote the class of *feasible* DTRs (for a more formal discussion of feasibility see (Schulte et al. 2014)). An optimal DTR, say *π*^opt^, satisfies $\mathbb {E}^{\pi ^{\text {opt}}}Y \ge \mathbb {E}^{\pi }Y$ for all *π*∈*Π*, where $\mathbb {E}^{\pi }$ denotes expectation under the restriction that *A*_*j*_=*π*_*j*_(*H*_*j*_). Define $Q_{2}(h_{2}, a_{2}) = \mathbb {E}(Y|H_{2}=h_{2}, A_{2}=a_{2})$ and $Q_{1}(h_{1}, a_{1}) = \mathbb {E}(\min _{a_{2}}Q_{2}(H_{2}, a_{2})|H_{1}=h_{1}, A_{1}=a_{1})$. The function *Q*_2_(*h*_2_,*a*_2_) measures the “quality” of assigning treatment *a*_2_ to a patient presenting at stage 2 with *H*_2_=*h*_2_; the function *Q*_1_(*h*_1_,*a*_1_) measures the quality of assigning treatment *a*_1_ to a patient presenting at stage 1 with *H*_1_=*h*_1_ assuming optimal subsequent treatment. It follows from dynamic programming (Bellman 1957) that $\pi _{j}^{\text {opt}}(h_{j}) = \arg \min _{a_{j}\in \mathcal {F}_{j}(h_{j})}Q_{j}(h_{j}, a_{j})$. In practice, dynamic programming cannot be applied because the true *Q*-functions are not known; instead, estimation of *π*^opt^ must rely on the observed data. *Q*-learning is an approximate dynamic programming algorithm which mimics the dynamic programming solution by replacing the conditional expectations required by dynamic programming with regression models fit to the observed data. Let *Q*_*j*_(*h*_*j*_,*a*_*j*_;*θ*_*j*_) denote a postulated working model for *Q*_*j*_(*h*_*j*_,*a*_*j*_) indexed by unknown parameter *θ*_*j*_.

In RAD, only patients who receive placebo as their first-stage treatment and failed to respond are randomized at the second stage. Thus, we only use these subjects to estimate *θ*_2_. Let *R* denotes a subject first-stage responder status so that *R*=1 for responders and *R*=0 for non-responders. Define 1_*u*_ to be equal to one if the condition *u* is true and zero otherwise. A version of the *Q*-learning algorithm that applies to data from RAD is:

**Algorithm 1: Q-learning for RAD**

**(Q1)** Compute $\widehat {\theta }_{2} = \arg \min _{\theta _{2}} \sum _{i=1}^{n}\{ Y_{i} - Q_{2}(H_{2i}, A_{2i};\theta _{2})\}^{2} 1_{A_{1i} = \text {placebo}}(1-R_{i})$; and subsequently estimator $Q_{2}\left (h_{2}, a_{2};\widehat {\theta }_{2}\right)$ of *Q*_2_(*h*_2_,*a*_2_).

**(Q2)** Define $\widehat {Y}_{i} = 1_{A_{1i} = \text {placebo}}\left (1-R_{i}\right) \min _{a_{2}\in \mathcal {F}_{2}(H_{2i})} Q_{2}\left (H_{2i}, a_{2};\widehat {\theta }_{2}\right) + (1_{A_{1i} \neq \text {placebo}} + R_{i}1_{A_{1i}=\text {placebo}}) Y_{i} $.

**(Q3)** Compute $\widehat {\theta }_{1} = \arg \min _{\theta _{1}} \sum _{i=1}^{n}\{ \widehat {Y}_{i} - Q_{1}(H_{1i}, A_{1i};\theta _{1})\}^{2}$ and subsequently estimator $Q_{1}\left (h_{1}, a_{1}; \widehat {\theta }_{1}\right)$ of *Q*_1_(*h*_1_,*a*_1_).

The *Q*-learning estimator of the optimal regime is $\widehat {\pi }_{j}\left (h_{j}\right) = \arg \min _{a_{j}\in \mathcal {F}_{j}(h_{j})}Q_{j}\left (h_{j}, a_{j};\widehat {\theta }_{j}\right)$. To estimate *π*^opt^ using data from the RAD study, we posit linear models for the *Q*-functions. For *Q*_1_(*h*_1_,*a*_1_), we posit a model of the form $Q_{1}(h_{1}, a_{1};\theta _{1}) = h_{10}^{\intercal }\beta _{10} + a_{11}h_{11}^{\intercal }\beta _{11} + a_{12}h_{12}^{\intercal }\beta _{12}$, where $\theta _{1} = \left (\beta _{10}^{\intercal }, \beta _{11}^{\intercal }, \beta _{12}^{\intercal }\right)^{\intercal }$, *h*_1*k*_, *k*=0,1,2 are known summary vectors of *h*_1_, and *a*_1*k*_,*k*=1,2 are dummy variables coding two of the three possible treatments at the first stage. For *Q*_2_(*h*_2_,*a*_2_), we posit a model of the form $Q_{2}\left (h_{2}, a_{2};\theta _{2}\right) = h_{20}^{\intercal }\beta _{20} + a_{2}h_{21}^{\intercal }\beta _{21}$, where $\theta _{2} = \left (\beta _{20}^{\intercal }, \beta _{21}^{\intercal }\right)^{\intercal }$, *h*_2*k*_, *k*=0,1 are known summary vectors of *h*_2_, and *a*_2_ is a dummy variable coding one of the two possible treatments at the second stage.

## Results

### Analysis of STEP-BD

The *Q*-learning algorithm stated in the preceding section assumes (i) complete data and (ii) working models for the *Q*-functions. However, in RAD, as in most clinical trials, a non-trivial amount of covariate and outcome information are missing. Furthermore, there is no strong theory to suggest working models for the *Q*-functions. So we must use the data to assist in the choice of these models. We combine multiple imputation with stepwise variable selection to estimate the *Q*-functions and subsequently the optimal treatment regime.

### Missing data

**Figure 3:**
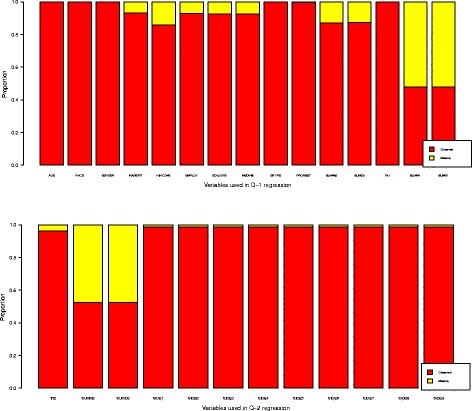
**Variable missingness.** Variables with missing data are listed. The SUMM*i* and SUMD*i* denote continuous symptom subscales for depression and mood elevation at *i*th stage. The Trt*i* denotes current treatment at stage *i*. The response*i* denotes patients’ clinical status at the end of stage *i*. The SIDE*j* represents different side effects. PRONSET denotes patients’ prior to onset clinical status. EDUCATE, EMPLOY, MARSTAT, MEDINS, and HINCOME are the indicators for patients’ education level, employment status, marriage status, medical insurance, and annual home income, respectively.

Figure 3 shows the proportions of missing values for the variables under consideration in our analysis of the RAD data. There is a significant amount of missing covariate information at both stages; thus, discarding subjects with missing information is inefficient and may introduce bias (Little and Rubin 2002).

One approach in dealing with missing data is multiple imputation (MI) (Rubin 2004). MI creates multiple complete datasets and is thereby suited for conducting a series of exploratory and secondary analyses including estimation of an optimal treatment regime (Shortreed et al. 2011). We use Bayesian MI to “fill in” the missing values which draws from the posterior predictive distribution of the missing values given the observed data (for details and underlying assumptions, see (Little and Rubin 2002; Van Buuren 2012)). Implementation of Bayesian MI requires specification of a prior and likelihood for the observed data. We specify the joint likelihood through the conditional distribution of each variable on all other variables (for discussion of this approach, see (Raghunathan et al. 2001; Van Buuren et al. 2006; Van Buuren 2007)). Thus, the likelihood is determined implicitly through a series of regression models, one for each variable that contains missing information. For continuous variables, we use predictive mean matching, and for binary variables, we use logistic regression models. To reduce variance, we use forward stepwise variable selection applied to the complete data to select predictors for each conditional model. Flat improper priors were used for all parameters. Imputations were carried out using the freely available and open-source mice package with the default settings (http://cran.r-project.org/web/packages/mice/index.html). Complete R code implementing our imputation model is provided in Additional file 1.

Using the procedure described above, we impute *m* complete datasets. For a given choice of *h*_1,*k*_, *k*=0,1,2 and *h*_2,*k*_, *k*=0,1, we can apply the *Q*-learning algorithm to each imputed dataset to obtain estimated *Q*-functions $Q_{j}\left (h_{j}, a_{j};\widehat {\theta }_{j}^{(\ell)}\right),\,j=1,2,\,\ell =1,\ldots, m$. The final estimated optimal decision rule is obtained as the minimizer of the averaged imputed *Q*-functions $\widehat {\pi }_{j}\left (h_{j}\right) = \arg \min _{a_{j}\in \mathcal {F}_{j}\left (h_{j}\right)} m^{-1}\sum _{\ell =1}^{m}Q_{j}\left (h_{j}, a_{j};\widehat {\theta }_{j}^{(\ell)}\right)$.

### Estimated optimal treatment regime and empirical results

**Figure 4:**
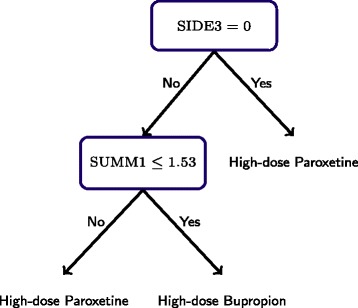
**Estimated optimal second-stage decision rule.** As anticipated by the estimated second-stage *Q*-function, SUMM1 (scale score for mood elevation) and SIDE3 (sedation side effect) are used to dictate treatment.

We use a version of stepwise variable selection to optimize the Bayesian information criteria (BIC); a complete description of this procedure is given in the Appendix section. The variables included in the model for the second-stage *Q*-function are SIDE3, SUMD1, and SUMM1. The variables included in the model for the first-stage *Q*-function are AGE, PRONSET, SUMD0, and SUMM0. Thus, the second-stage *Q*-functions has the form $Q_{2}\left (h_{2},a_{2};\theta _{2}\right) = \beta _{20}^{\intercal } h_{20} + a_{2} \beta _{21}^{\intercal } h_{21}$, where $h_{20} = (1,SUMM1, SUMD1, SIDE3)^{\intercal },\, h_{21} = (1,SUMM1, SIDE3)^{\intercal },$ and *A*_2_ is indicator variable for stage 2 treatment coded so that *A*_2_=1 denotes high-dose Bupropion and *A*_2_=0 denotes high-dose Paroxetine. The estimated coefficients $\widehat {\beta }_{20}, \widehat {\beta }_{21}$ along with 90% bootstrap confidence intervals are shown in Table 2. The table shows that the main effect of *A*_2_ and interaction between second *A*_2_ and SUMM1 is significant at the 90% level. The estimated optimal decision rule is shown in Figure 4. As anticipated by estimated second-stage *Q*-function, SUMM1 (mood severity) and SIDE3 (sedation side effect) dictate treatment selection; subjects with sedation side effects and low mood severity are recommended to Bupropion, and all others are recommended to Paroxetine.

**Table 2:** **Point estimates and confidence intervals for the coefficients indexing the second-stage** ***Q*** **-function**

**Variable**	**Abbreviation**	**Coefficient**	**90% confidence**
		**estimate**	**interval**
SUMM1	SUMM1	0.18	(−0.14, 0.90)
SUMD1	SUMD1	0.50	(0.48, 0.83)
Side 3	SIDE3	−0.41	(−2.65, 0.42)
Intercept	Int	2.21	(0.05, 2.00)
*A*_2_×SUMM1	A2 _SUMM1	0.77	(−0.16, 1.18)
*A*_2_×Side 3	A2 _SIDE3	1.82	(−1.05, 3.18)
*A*_2_	A2	−1.18	(−1.98, 0.00)

The first-stage *Q*-function has the form $Q_{1}(h_{1}, a_{1};\theta _{1}) = \beta _{10}^{\intercal } h_{10} + a_{11} \beta _{11}^{\intercal } h_{11} + a_{12} \beta _{12}^{\intercal } h_{12}$, where:

$${\small{ \begin{aligned} h_{10} &= (1,\text{AGE}, \text{SUMM0}, \text{SUMD0}, \text{PRONSET1}, \text{PRONSET2})^{\intercal};\\ h_{11} &= (1, \text{SUMM0}, \text{PRONSET1}, \text{PRONSET2})^{\intercal}; \\ h_{12} &= (1, \text{SUMM0}, \text{PRONSET1}, \text{PRONSET2})^{\intercal}; \end{aligned}}} $$

**Figure 5:**
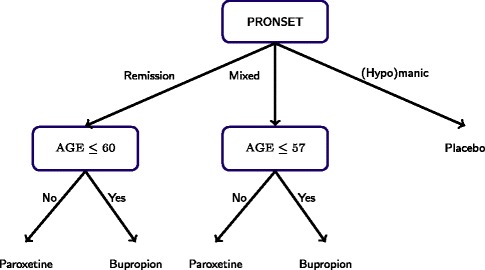
**Estimated optimal first-stage decision rule.** Note that subjects with (hypo)manic episodes immediately preceding the current major depressive episode are recommended to receive placebo.

*a*_11_=1 if *a*_1_=Bupriopion otherwise *a*_11_=0; *a*_12_=1 if *a*_1_=Paroxetine otherwise *a*_12_=0; PRONSET1=1 if PRONSET=remission otherwise PRONSET1=0; and PRONSET2=1 if PRONSET=manic or hypomanic otherwise PRONSET2=0. The estimated coefficients and 90% bootstrap intervals (corrected for non-regularity as suggested by (Chakraborty et al. 2013)) are listed in Table 3. Figure 5 shows the first-stage optimal decision rule implied by the estimated *Q*-function. An interesting feature of the first-stage decision rule is that subjects with a (hypo)manic episode immediately preceding the current major depressive episode are recommended to receive placebo. This supports the hypothesis that subjects with (hypo)manic episodes immediately preceding a major depressive episode might not benefit from an adjuvant antidepressant. Figure 5 also shows that among the subjects experiencing remission or mixed/cycling before the current major depressive episodes, Bupropion is recommended to older patients and Paroxetine is recommended to younger patients.

**Table 3:** **Point estimates and confidence intervals for the coefficients indexing the first-stage** ***Q*** **-function**

**Variable**	**Abbreviation**	**Coefficient**	**90% Confidence**
		**estimate**	**interval**
Age	AGE	0.02	(−0.01, 0.04)
SUMM0	SUMM0	0.48	(0.35, 0.70)
SUMD0	SUMD0	0.20	(0.15, 0.36)
Prior episode 1	PRONSET1	−0.42	(−1.07, 0.42)
Prior episode 2	RPOSNET2	−0.86	(−1.49, -0.05)
Intercept	Int	1.57	(−0.49, 2.50)
*A*_11_×AGE	A11 _AGE	0.01	(−0.04, 0.07)
*A*_11_×PRONSET1	A11 _PRONSET1	0.66	(−0.80, 2.29)
*A*_11_×PRONSET2	A11 _PRONSET2	1.13	(−0.55, 2.82)
*A*_11_	A11	−1.55	(−4.07, 1.25)
*A*_12_×AGE	A12 _AGE	−0.03	(−0.08, 0.02)
*A*_12_×PRONSET1	A12 _PRONSET1	0.79	(−0.51, 1.92)
*A*_12_×PRONSET2	A12 _PRONSET2	1.62	(0.05, 2.90)
*A*_12_	A12	0.73	(−1.48, 3.08)

Recall that the optimal treatment regime minimizes the expected depression score SUMD measured at week 12. Thus, it is of interest to compare the estimated expected 12-week SUMD under the estimated optimal treatment regime and other potential treatment regimes of interest. Table 4 shows the estimated depression score under the estimated regime and four static treatment regimes. Estimates were computed using the inverse-probability-weighted estimator (IPWE) (Zhang et al. 2013) and confidence intervals using the non-parametric bootstrap. The estimated optimal regime performs significantly better at the 90% level than any fixed regime under consideration. We note, however, that the confidence intervals must be interpreted with caution as the IPWE is not a smooth estimator which may cause the bootstrap to perform poorly (note that normal approximations do not hold either without strong assumptions (Laber and Murphy 2011)).

**Table 4:** **Point estimates and confidence intervals for the expected depression score SUMD at week 12 under static regimes (first-line treatment, second-line treatment) and estimated DTR**

**Regime (** ***π***_**1**_ **,** ***π***_**2**_ **)**	**Estimated**	**90% Confidence**
	**SUMD**	**interval**
Estimated DTR	2.13	(1.34, 2.86)
(Bupropion, high-dose Bupropion)	6.91	(6.27, 7.71)
(Paroxetine, high-dose Paroxetine)	8.25	(7.39, 9.07)
(placebo, high-dose Bupropion)	3.71	(3.38, 4.04)
(placebo, high-dose Paroxetine)	4.51	(4.10, 4.90)

## Discussion

We estimated an optimal DTR for patients presenting with bipolar depression using data from the RAD pathway in the STEP-BD study. The estimated treatment regime suggests the hypothesis that bipolar-depression patients with (hypo)mania immediately preceding a major depressive episode may do better to forgo adjunctive antidepressant treatment with either paroxetine or bupropion, whereas the opposite is true for who were in remission or experienced a mixed episode (manic episode with mixed features, according to DSM-V) before the current major depressive episode. This is a novel finding, which has not been explored so far. At present, there is a consensus that antidepressants in the acute treatment of bipolar depression may be used when there is a history of previous positive response to antidepressants, while they should be avoided in patients with an acute bipolar depressive episode with two or more concomitant core manic symptoms in the presence of psychomotor agitation, in patients with a high number of previous episodes or with a history of rapid cycling and during depressive episodes with mixed features (Pacchiarotti et al. 2013). Furthermore, the use of antidepressants is discouraged if there is a history of past mania, hypomania, or mixed episodes emerging during antidepressant treatment (Pacchiarotti et al. 2013). However, this consensus is mainly based on clinical wisdom than strong external evidence. In our study, the scale scores for measuring symptoms of depression as well as mania were available for baseline and stage 1 to model the *Q*-functions but did not turn out to be helpful in building an optimal DTR.

So far, there are no reliable data on the differential efficacy of paroxetine and bupropion in younger or older adult patients with bipolar depression. In unipolar depression, a recent meta-analysis suggests that the efficacy of antidepressants in general may be reduced in trials involving patients aged 65 years or older (Tedeschini et al. 2011). Similarly, there have not been any reliable data suggesting that patients with higher scores on mood elevation scales do better on paroxetine than bupropion—and vice versa (Pacchiarotti et al. 2013). What is well known on the other hand is that Paroxetine 20 mg/day does not seem to be associated with an increased risk of switch into (hypo)mania in patients with bipolar depression, even in monotherapy (McElroy et al. 2010). The data for our analyses stem from a double-blind, randomized, placebo-controlled trial (Sachs et al. 2007). Consequently, we do not know whether in clinical practice not adding any medication or intervention to a mood stabilizer is of comparable benefit for those who do best on placebo in our analyses (Severus et al. 2012).

## Conclusions

As mentioned in the introduction, estimation of an optimal DTR is typically done as a secondary, exploratory analysis and viewed as a method of generating hypotheses for follow-up confirmatory experiments. The latter is just about to start, using patients with bipolar depression being openly treated within the SCP pathway of STEP-BD using the same rating forms, in particular, the clinical monitoring form for mood disorders.

## Appendix

### Variable selection

In order to estimate an optimal treatment regime using *Q*-learning, we need to select which covariates to include the models for the *Q*-functions. Recall that the 12-week depression SUMD score was used as the outcome (*Y*). We identified 24 potential predictors; these predictors are listed in Table 5. We select a subset of these predictors for each *Q*-function using stepwise variable selection to minimize the Bayes information criterion (BIC) (Schwarz et al. 1978) averaged over the multiply imputed datasets. Let $\mathcal {M}_{2}$ denote a subset of predictors dictating the features *h*_2,*k*_, *k*=0,1, and let $\widehat {\theta }_{2}^{(\ell)}(\mathcal {M}_{2})$ denote the coefficients obtained by applying step (Q1) of the *Q*-learning algorithm with predictors $\mathcal {M}_{2}$ to the *ℓ*th imputed dataset. Define $\widehat {Y}_{i}^{(\ell)}(\mathcal {M}_{2}), \,i=1,\ldots, n$ to be the predicted outcomes computed in step (Q2) of the *Q*-learning algorithm using the *ℓ*th imputed dataset and parameter $\widehat {\theta }_{2}^{(\ell)}(\mathcal {M}_{2})$. Let $\mathcal {M}_{1}$ denotes a subset of predictors dictating *h*_1,*k*_, *k*=0,1,2, and let $\widehat {\theta }_{1}^{(\ell)}(\mathcal {M}_{1}, \mathcal {M}_{2})$ denote the coefficients estimated in step (Q3) of the *Q*-learning algorithm using predictors $\mathcal {M}_{1}$ and predicted outcomes $\widehat {Y}_{i}^{(\ell)}(\mathcal {M}_{2}), \,i=1,\ldots, n$. In addition, let $\text {BIC}^{(\ell)}\left (\mathcal {M}_{2}\right)$ denote the BIC for a second-stage model $Q\left (h_{2}, a_{2};\widehat {\theta }_{2}^{(\ell)}(\mathcal {M}_{2})\right)$ calculated on the *ℓ*th imputed dataset. Similarly, and let $\text {BIC}^{(\ell)}(\mathcal {M}_{1}, \mathcal {M}_{2})$ denote the BIC for the first-stage model $Q\left (h_{1}, a_{1};\widehat {\theta }_{1}^{(\ell)}(\mathcal {M}_{1}, \mathcal {M}_{2})\right)$ calculated on the *ℓ*th imputed dataset. This procedure that we use to construct models for the *Q*-learning algorithm is:

- Using forward variable selection compute:
  $$ \widehat{\mathcal{M}}_{2} = \arg\min_{\mathcal{M}_{2}}\frac{1}{m} \sum_{\ell=1}^{m}\text{BIC}^{(\ell)}(\mathcal{M}_{2}); $$

**Table 5:** **Candidate predictors for regression models in** ***Q*** **-learning**

**Variable**	**Description**	**Abbreviation**	**Type**	**Values (range or level)**	**Mean (SD) or frequency (** ***%*** **)**
Age	Age at entry (years)	AGE	Numerical	18–77	40.59 (11.74)
Race	Race	RACE	Binary	White or Causasian, non-White	90.4*%*, 9.6*%*
Gender	Gender	GENDER	Trinary	Male, female, transgender	43*%*, 56*%*, 1*%*
Marriage	Marital status	MARSTAT	Trinary	Never married, married,	35.6*%*, 33.8*%*, 30.6*%*
				separated	
Household Income	Annual household income	HINCOME	Binary	<40, ≥40	58.5*%*, 41.5*%*
	(×$1000)				
Employment	Employment status	EMPLOY	Binary	Employed, unemployed	46.9*%*, 53.1*%*
Education	Education level	EDUCATE	Binary	College or below, technical	53*%*, 47*%*
				school or above	
Insurance	Indicator of medical insurance	MEDINS	Binary	Yes, no	72.8*%*, 27.2*%*
Bipolar Type	Bipolar type at entry	BITYPE	Binary	Type I, type II	70.4*%*, 29.6*%*
Prior Episode	Clinical episode immediately	PRONSET	Trinary	Remission, (hypo)manic,	45.9*%*, 33.2*%*, 20.9*%*
	preceding current depressive			mixed	
	episode				
SUMD0	Scaled depression at entry	SUMD0	Numerical	0.75–18	7.47 (2.30)
SUMD1 ^a^	Scaled depression at the end	SUMD1	Numerical	0–14	4.49 (3.07)
	of stage 1				
SUMM0	Scaled mood elevation at entry	SUMM0	Numerical	0–7	1.19 (1.09)
SUMM1 ^a^	Scaled mood elevation at the	SUMM1	Numerical	0–6.75	0.95 (1.30)
	end of stage 1				
Treatment 1 ^a^	Treatment received at stage 1	Trt1	Trinary	Bupropion, Paroxetine,	23.3*%*, 25.5*%*, 51.2*%*
				placebo	
Side 1	Tremor	SIDE1	Binary	Yes, no	26.9*%*, 73.1*%*
Side 2	Dry mouth	SIDE2	Binary	Yes, no	21.1*%*, 78.9*%*
Side 3	Sedation	SIDE3	Binary	Yes, no	17.1*%*, 82.9*%*
Side 4	Constipation	SIDE4	Binary	Yes, no	5.7*%*, 94.3*%*
Side 5	Diarrhea	SIDE5	Binary	Yes, no	12*%*, 88*%*
Side 6	Headache	SIDE6	Binary	Yes, no	13.7*%*, 86.3*%*
Side 7	Poor memory	SIDE7	Binary	Yes, no	14.3*%*, 85.7*%*
Side 8	Sexual dysfunction	SIDE8	Binary	Yes, no	9.7*%*, 90.3*%*
Side 9	Increased appetite	SIDE9	Binary	Yes, no	12.6*%*, 87.4*%*

^a^Those that are only available for the second-stage regression model.
- Using forward variable selection compute:
  $$ \widehat{\mathcal{M}}_{1} = \arg\min_{\mathcal{M}_{1}}\frac{1}{m} \sum_{\ell=1}^{m}\text{BIC}^{(\ell)}\left(\mathcal{M}_{1}, \widehat{\mathcal{M}}_{2}\right); $$
- Let $$ {Q}_2\kern0.3em \left({h}_2,\kern0.3em {a}_2\kern0.3em ;{\hat{\theta}}_2\kern0.3em \left({\hat{\mathcal{M}}}_2\kern0.3em \right)\kern0.3em \right) $$ and $Q_{1}\!\left (\!h_{1}\!, a_{2};\widehat {\theta }_{1}\left (\!\widehat {\mathcal {M}}_{1}, \widehat {\mathcal {M}}_{2}\!\right)\right)$ denote the second- and first-stage estimated *Q*-functions, respectively.

## Additional file

Additional file 1
**Multiple Imputation for Missing Data in RAD Data.**

## References

[CR1] Bauer M, Pfennig A, Severus E, Whybrow PC, Angst J, Möller H-J (2013). World federation of societies of biological psychiatry (wfsbp) guidelines for biological treatment of unipolar depressive disorders, part 1: update 2013 on the acute and continuation treatment of unipolar depressive disorders. World J Biol Psychiatry.

[CR2] Bellman RE (1957). Dynamic programming.

[CR3] Chakraborty B, Murphy SA (2014). Dynamic treatment regimes. Annu Rev Stat Appl.

[CR4] Chakraborty B, Laber EB, Zhao Y (2013). Inference for optimal dynamic treatment regimes using an adaptive m-out-of-n bootstrap scheme. Biometrics.

[CR5] Chakraborty B, Moodie EE (2013). Statistical reinforcement learning. Statistical methods for dynamic treatment regimes.

[CR6] Goldberg Y, Kosorok MR (2012). Q-learning with censored data. Ann Stat.

[CR7] Grunze H, Vieta E, Goodwin GM, Bowden C, Licht RW, Möller H-J (2010). The World Federation of Societies of Biological Psychiatry (WFSBP) guidelines for the biological treatment of bipolar disorders: update 2010 on the treatment of acute bipolar depression. World J Biol Psychiatry.

[CR8] Kleine-Budde K, Touil E, Moock J, Bramesfeld A, Kawohl W, Rössler W (2014). Cost of illness for bipolar disorder: a systematic review of the economic burden. Bipolar Disord.

[CR9] Laber EB, Lizotte DJ, Qian M, Pelham WE, Murphy SA (2014). Dynamic treatment regimes: Technical challenges and applications. Electron J Stat.

[CR10] Laber EB, Lizotte DJ, Ferguson B (2014). Set-valued dynamic treatment regimes for competing outcomes. Biometrics.

[CR11] Laber EB, Murphy SA (2011). Adaptive confidence intervals for the test error in classification. J Am Stat Assoc.

[CR12] Lavori PW, Dawson R (2004). Dynamic treatment regimes: practical design considerations. Clin Trials.

[CR13] Leboyer M, Kupfer DJ (2010). Bipolar disorder: new perspectives in health care and prevention. J Clin Psychiatry.

[CR14] Lei H, Nahum-Shani I, Lynch K, Oslin D, Murphy S (2012). A “smart” design for building individualized treatment sequences. Annu Rev Clin Psychol.

[CR15] Licht R, Gijsman H, Nolen W, Angst J (2008). Are antidepressants safe in the treatment of bipolar depression? A critical evaluation of their potential risk to induce switch into mania or cycle acceleration. Acta Psychiatr Scand.

[CR16] Little, RJA, Rubin DB. Statistical analysis with missing data (second Edition): Chichester: Wiley; 2002.

[CR17] McElroy SL, Weisler RH, Chang W, Olausson B, Paulsson B, Brecher M (2010). A double-blind, placebo-controlled study of quetiapine and paroxetine as monotherapy in adults with bipolar depression (embolden ii). J Clin Psychiatry.

[CR18] Moodie EE, Dean N, Sun YR (2014). Q-learning: flexible learning about useful utilities. Stat Biosci.

[CR19] Moodie EE, Richardson TS, Stephens DA (2007). Demystifying optimal dynamic treatment regimes. Biometrics.

[CR20] Murphy SA (2003). Optimal dynamic treatment regimes (with discussion). J R Stat Soc.

[CR21] Murphy, SA (2005). An experimental design for the development of adaptive treatment strategies. Stat Med.

[CR22] Murphy, SA (2005). A generalization error for Q-learning. J Mach Learn Res: JMLR.

[CR23] Nahum-Shani I, Qian M, Almirall D, Pelham WE, Gnagy B, Fabiano GA (2012). Experimental design and primary data analysis methods for comparing adaptive interventions. Psychol Methods.

[CR24] Qian M, Almirall D, Pelham WE, Gnagy B, Fabiano GA, Nahum-Shani, I (2012). Q-learning: a data analysis method for constructing adaptive interventions. Psychol Methods.

[CR25] Nierenberg AA, Friedman ES, Bowden CL, Sylvia LG, Thase ME, Ketter T (2013). Lithium treatment moderate-dose use study (LiTMUS) for bipolar disorder: a randomized comparative effectiveness trial of optimized personalized treatment with and without lithium. Am J Psychiatry.

[CR26] Pacchiarotti I, Bond DJ, Baldessarini RJ, Nolen WA, Grunze H, Licht RW (2013). The International Society for Bipolar Disorders (ISBD) task force report on antidepressant use in bipolar disorders. Am J Psychiatry.

[CR27] Phillips ML, Kupfer DJ (2013). Bipolar disorder diagnosis: challenges and future directions. The Lancet.

[CR28] Raghunathan TE, Lepkowski JM, Van Hoewyk J, Solenberger P (2001). A multivariate technique for multiply imputing missing values using a sequence of regression models. Surv Methodol.

[CR29] Robins JM (2004). Optimal structural nested models for optimal sequential decisions. Proceedings of the second seattle symposium in biostatistics.

[CR30] Rubin, DB. Multiple imputation for nonresponse in surveys *(Vol. 81)*: John Wiley & Sons; 2004.

[CR31] Sachs GS, Thase ME, Otto MW, Bauer M, Miklowitz D, Wisniewski SR (2003). Rationale, design, and methods of the systematic treatment enhancement program for bipolar disorder (step-bd). Biol Psychiatry.

[CR32] Sachs GS, Nierenberg AA, Calabrese JR, Marangell LB, Wisniewski SR, Gyulai L (2007). Effectiveness of adjunctive antidepressant treatment for bipolar depression. N Engl J Med.

[CR33] Sachs GS, Guille C, McMurrich SL (2002). A clinical monitoring form for mood disorders. Bipolar Disord.

[CR34] Schwarz G (1978). Estimating the dimension of a model. Ann Stat.

[CR35] Schulte PJ, Tsiatis AA, Laber EB, Davidian M (2014). Q-and A-learning methods for estimating optimal dynamic treatment regimes. Stat Sci: Rev J Inst Math Stat.

[CR36] Severus E, Seemüller F, Berger M, Dittmann S, Obermeier M, Pfennig A (2012). Mirroring everyday clinical practice in clinical trial design: a new concept to improve the external validity of randomized double-blind placebo-controlled trials in the pharmacological treatment of major depression. BMC Medicine.

[CR37] Shortreed SM, Laber E, Lizotte DJ, Stroup TS, Pineau J, Murphy SA (2011). Informing sequential clinical decision-making through reinforcement learning: an empirical study. Mach Learn.

[CR38] Sterne JA, May M, Costagliola D, De Wolf F, Phillips AN, Harris R (2009). Timing of initiation of antiretroviral therapy in AIDS-free HIV-1-infected patients: a collaborative analysis of 18 HIV cohort studies. The Lancet.

[CR39] Strecher VJ, Shiffman S, West R (2006). Moderators and mediators of a web-based computer-tailored smoking cessation program among nicotine patch users. Nicotine Tobacco Res.

[CR40] Sutton, RS, Barto AG. Reinforcement learning: an introduction: MIT press; 1998.

[CR41] Tedeschini E, Levkovitz Y, Iovieno N, Ameral VE, Nelson JC, Papakostas GI (2011). Efficacy of antidepressants for late-life depression: a meta-analysis and meta-regression of placebo-controlled randomized trials. J Clin Psychiatry.

[CR42] Van Buuren S, Brand JP, Groothuis-Oudshoorn C, Rubin DB (2006). Fully conditional specification in multivariate imputation. J Stat Comput Simul.

[CR43] Van Buuren S (2007). Multiple imputation of discrete and continuous data by fully conditional specification. Stat Methods Med Res.

[CR44] Van Buuren, S. Flexible imputation of missing data: CRC press; 2012.

[CR45] Vos T, Flaxman AD, Naghavi M, Lozano R, Michaud C, Ezzati M (2013). Years lived with disability (YLDs) for 1160 sequelae of 289 diseases and injuries 1990–2010: a systematic analysis for the Global Burden of Disease Study 2010. The Lancet.

[CR46] Watkins CJ, Dayan P (1992). Q-learning. Mach Learn.

[CR47] Wagner EH, Austin BT, Davis C, Hindmarsh M, Schaefer J, Bonomi A (2001). Improving chronic illness care: translating evidence into action. Health Aff.

[CR48] Zhang B, Tsiatis AA, Laber EB, Davidian M (2013). Robust estimation of optimal dynamic treatment regimes for sequential treatment decisions. Biometrika.

