# Supplementary material for: Who will benefit from antidepressants in the acute treatment of bipolar depression? A reanalysis of the STEP-BD study by Sachs et al. 2007, using Q-learning
Source: Int J Bipolar Disord. 2015 Apr 3;3:7. doi: 10.1186/s40345-014-0018-5 (PMC4383759; doi:10.1186/s40345-014-0018-5)
Supplement: Additional file 1 — Multiple Imputation for Missing Data in RAD Data. [file 40345_2014_18_MOESM1_ESM.pdf]

Multiple Imputation for Missing Data in RAD Data

There are 366 patients in RAD. A significant amount of missing covariate information are occurred in both stage. So we use Multiple Imputation (MI) to deal with the missing data. We use open source *mice* package in software R. After the procedure of MI, 5 complete data sets are created. Below is the R code we used for this whole MI procedure.

```
#################################################################
###### Missing Data Imputation, Fan Wu       ###################
###### m = 5, predictorMatrix var number = 5 ###################
###### Via R software
#################################################################


rm(list=ls())
#setwd("//stat.ad.ncsu.edu/Redirect/fwu5/Desktop/STEP-BD_V2/RAD")  # set directory
setwd("E:/NCSU_ST/Research/STEP-BD_V2/RAD")
raw_data <- read.csv("clean_2_data.csv", header=TRUE)
raw_data <- raw_data[1:365,]

library(mice)
#################################################################
##### recode the variables (decrease DF of each variable)########
names(raw_data)  # list all the var names
## using index to extract some index
index = which(!(raw_data$Race==1))
raw_data$Race[index] = 0
index = which((raw_data$MARSTAT %in% c(1)))
raw_data$MARSTAT[index] = 1
index = which((raw_data$MARSTAT %in% c(2,3)))
raw_data$MARSTAT[index] = 2
index = which((raw_data$MARSTAT %in% c(4,5,6)))
raw_data$MARSTAT[index] = 3
index = which(raw_data$HINCOME %in% c(1,2,3,4))
raw_data$HINCOME[index] = 0
index = which(raw_data$HINCOME %in% c(5,6,7,8,9,10,11))
raw_data$HINCOME[index] = 1
index = which((raw_data$EMPLOY %in% c(1,2)))
raw_data$EMPLOY[index] = 1
index = which(raw_data$EMPLOY %in% c(3,5,6,7,8,9))
raw_data$EMPLOY[index] = 0
index = which(raw_data$EDUCATE %in% c(1,2,3,4,5))
raw_data$EDUCATE[index] = 0
index = which(raw_data$EDUCATE %in% c(6,7,8))
raw_data$EDUCATE[index] = 1
index = which(raw_data$response_1 %in% c(2))
raw_data$response_1[index] = 0
```

```
#index = which(raw_data$response_2 %in% c(2))
#raw_data$response_2[index] = 0
index = which(raw_data$response_1 %in% c(3,4))
raw_data$response_1[index] = 2
#index = which(raw_data$response_2 %in% c(1,3,4))
#raw_data$response_2[index] = 1
index = which(!(raw_data$SIDE_1 ==0))
raw_data$SIDE_1[index] = 1
index = which(!(raw_data$SIDE_2 ==0))
raw_data$SIDE_2[index] = 1
index = which(!(raw_data$SIDE_3 ==0))
raw_data$SIDE_3[index] = 1
index = which(!(raw_data$SIDE_4 ==0))
raw_data$SIDE_4[index] = 1
index = which(!(raw_data$SIDE_5 ==0))
raw_data$SIDE_5[index] = 1
index = which(!(raw_data$SIDE_6 ==0))
raw_data$SIDE_6[index] = 1
index = which(!(raw_data$SIDE_7 ==0))
raw_data$SIDE_7[index] = 1
index = which(!(raw_data$SIDE_8 ==0))
raw_data$SIDE_8[index] = 1
index = which(!(raw_data$SIDE_9 ==0))
raw_data$SIDE_9[index] = 1


# Before doing Multiple Imputation, check the correlations
MI_data1 = raw_data[,c(-1,-2,-13,-22)]


corr_matrix = cor(MI_data1, use="pairwise.complete.obs", method="pearson")
abs_corr = abs(corr_matrix)
var_name = rownames(corr_matrix)

## record the 5 highest correlation variables for each one
impute_var_index = matrix(0, 5, length(var_name))

for(i in 1:length(var_name))
{
  impute_var_index[,i] = order(abs_corr[i,],decreasing = TRUE)[2:6]
}

# define the highly correlated variables without second stage information:
impute_var_index[,16] = c(15,23,24,4,27)



# redefine the variable type
raw_data$STEPID = as.character(raw_data$STEPID)
```

```
raw_data$PathWay = as.character(raw_data$PathWay)
raw_data$Race = as.factor(raw_data$Race)
raw_data$Gender = as.factor(raw_data$Gender)
raw_data$MARSTAT = as.factor(raw_data$MARSTAT)
raw_data$HINCOME = as.factor(raw_data$HINCOME)
raw_data$EMPLOY = as.factor(raw_data$EMPLOY)
raw_data$EDUCATE = as.factor(raw_data$EDUCATE)
raw_data$MEDINS = as.factor(raw_data$MEDINS)
raw_data$BiTYPE = as.factor(raw_data$BiTYPE)
raw_data$PRONSET = as.factor(raw_data$PRONSET)
raw_data$SITE_ID = as.character(raw_data$SITE_ID)
raw_data$trt_1 = as.factor(raw_data$trt_1)
#raw_data$trt_2 = as.factor(raw_data$trt_2)
raw_data$response_1 = as.factor(raw_data$response_1)
raw_data$response_2 = as.factor(raw_data$response_2)
raw_data$SIDE_1 = as.factor(raw_data$SIDE_1)
raw_data$SIDE_2 = as.factor(raw_data$SIDE_2)
raw_data$SIDE_3 = as.factor(raw_data$SIDE_3)
raw_data$SIDE_4 = as.factor(raw_data$SIDE_4)
raw_data$SIDE_5 = as.factor(raw_data$SIDE_5)
raw_data$SIDE_6 = as.factor(raw_data$SIDE_6)
raw_data$SIDE_7 = as.factor(raw_data$SIDE_7)
raw_data$SIDE_8 = as.factor(raw_data$SIDE_8)
raw_data$SIDE_9 = as.factor(raw_data$SIDE_9)
raw_data$DRUG_NAME = as.factor(raw_data$DRUG_NAME)

MI_data = raw_data[,c(-1,-2,-13,-22)]

# construct imputation model
predictor_matrix = matrix(0, length(MI_data), length(MI_data))
for (i in 1:length(MI_data))
{
  predictor_matrix[i,impute_var_index[,i]]=rep(1,5)
}

MI = mice(MI_data, m = 5, predictorMatrix = predictor_matrix )

# output the imputation data set and save them as data1-data5:

data1 = data2 = data3 = data4 = data5 =MI_data
data = list(data1,data2,data3,data4,data5)
for(i in 1:5)
{
  Data = as.data.frame(data[i])
  for (j in c(4:8,10:12,14:27))
  {
    Data_impute = as.data.frame(MI$imp[j] )
    index = which(is.na(Data[j]) )
    var = Data[j]
```

```
  var[index,1] = Data_impute[,i]
  # index1 = which(is.na(Data[j]) & Data$response_1==1)
  # if (length(index1)>0) { var[index1,1] = NA}  # delete the R=1 imputation
  Data[j] = var
}

Data$Gender = as.numeric(Data$Gender)
Data$MARSTAT = as.numeric(Data$MARSTAT)

# Data$EDUCATE = as.numeric(Data$EDUCATE)

Data$BiTYPE = as.numeric(Data$BiTYPE)
Data$PRONSET = as.numeric(Data$PRONSET)


index = which((Data$BiTYPE %in% c(1)))
Data$BiTYPE[index] = 0
index = which((Data$BiTYPE %in% c(2)))
Data$BiTYPE[index] = 1

Data$Gender2 = Data$Gender1=rep(0,365)
index = which((Data$Gender %in% c(1)))
Data$Gender1[index] = 1
index = which((Data$Gender %in% c(2)))
Data$Gender2[index] = 1

Data$Mariage2 = Data$Mariage1=rep(0,365)
index = which((Data$MARSTAT %in% c(1)))
Data$Mariage1[index] = 1
index = which((Data$MARSTAT %in% c(2)))
Data$Mariage2[index] = 1


Data$PRONSET2 = Data$PRONSET1=rep(0,365)
index = which((Data$PRONSET %in% c(1)))
Data$PRONSET1[index] = 1
index = which((Data$PRONSET %in% c(2)))
Data$PRONSET2[index] = 1




A_1 = Data$trt_1
A11 = A12  = rep(0,365)
index = which((A_1 %in% c(1)))
A11[index] = 1
index = which((A_1 %in% c(2)))
A12[index] = 1

Data$A11 = A11
```

```
  Data$A12 = A12

  A_2 = Data$DRUG_NAME
  A2 = rep(0,365)
  index = which((A_2 %in% c(1)))
  A2[index] = 1


  Data$A2 =A2


  data[i] = list(Data)
}


write.csv(data[1],"data1.csv")
write.csv(data[2],"data2.csv")
write.csv(data[3],"data3.csv")
write.csv(data[4],"data4.csv")
write.csv(data[5],"data5.csv")
```
